# Influence of Innate Immunity on Cancer Cell Stemness

**DOI:** 10.3390/ijms21093352

**Published:** 2020-05-09

**Authors:** Anna Pastò, Francesca Maria Consonni, Antonio Sica

**Affiliations:** 1 Department of Inflammation and Immunology, Humanitas Clinical and Research Center–IRCCS–, via Manzoni 56, 20089 Rozzano (MI), Italy; anna.pasto@humanitasresearch.it; 2 Department of Pharmaceutical Sciences, University of Eastern Piedmont, A. Avogadro, via Bovio 6, 28100 Novara, Italy; Francesca_Maria.Consonni@humanitasresearch.it

**Keywords:** cancer stem cells, innate immunity, tumor-associated macrophages, myeloid-derived suppressor cells, inflammation

## Abstract

Even if cancer stem cells (CSCs) represent only a small proportion of the tumor mass, they significantly account for tumor maintenance, resistance to therapies, relapse and metastatic spread, due to their increased capacity of self-renewal, multipotency, tumorigenicity and quiescence. Emerging evidence suggests that the immune contexture within the tumor microenvironment (TME) determines both the response to therapy and the clinical outcome. In this context, CSCs acquire immune evasion skills by editing immune cell functions and sculpting the immunosuppressive landscape of TME. Reciprocally, infiltrating immune cells influence CSCs self-renewal, tumorigenicity and metastasis. In this review, we summarize the immunomodulatory properties of CSCs, as well as the impact of innate immune cells on cancer cells stemness in the different phases of cancer immunoediting process and neoplastic progression.

## 1. Introduction

### 1.1. Cancer

The transformation of normal cells into a malignant tumor is a multistep process through which transforming cells acquire malignant features, described as “hallmarks of cancer”. These include sustaining proliferative signaling, evading anti-proliferative safeguards, resisting apoptotic programming, enabling replicative immortality, inducing angiogenesis, and activating invasion and metastasis. Underlying these hallmarks are genome instability and smoldering inflammation, which foster multiple functions of cancer cells [1]. Furthermore, new observations indicate that the changes to which the transformed cells are subjected, including their heterogeneity and stemness, are affected by and mutually influence the host’s immune-inflammatory response, suggesting a model of tumor/host interdependence, in which the determinants of neoplastic progression are still largely unclear.

### 1.2. Innate Immune Populations in Cancer

Solid tumors are composed not only of malignant cells, but are a complex network of heterogeneous cell populations, including fibroblasts, endothelial cells and leukocytes, engaged in reciprocal interactions guiding the construction of a permissive microenvironment for tumor growth. This complexity creates a physical network, the tumor microenvironment (TME), which gradually reprograms immune and micro-physiological responses towards conditions that promote tumor growth and metastasis [2,3].

Within this scenario, innate immune cells, i.e. macrophages (TAMs), neutrophils (TANs), dendritic cells (DCs), myeloid-derived suppressor cells (MDSCs) and natural killer cells (NK), are the key drivers of cancer-related inflammation and, due to their functional plasticity, can act decisive pro- or anti-tumorigenic roles during different stages of neoplastic progression. In fact, innate immunity can either block tumor development, by destroying tumor cells and/or inhibiting their growth, or support proliferation and survival of transformed cells, by sculpting their immunogenicity and/or inhibiting host’s protective anti-tumor responses [4,5,6,7].

This dynamic process has been conveyed in the “cancer immunoediting” hypothesis, encompassing three key events: the “Elimination” phase that corresponds to cancer immunosurveillance, where mostly tumor cells are detected and killed by components of the immune system; the “Equilibrium” phase, in which a balance is established between immune and cancer cells; the “Escape” phase, in which activation of immunosuppressive circuits allows immuno-evasion and spreading of cancer cells [8,9].

### 1.3. Cancer Stem Cells

**Figure 1:**
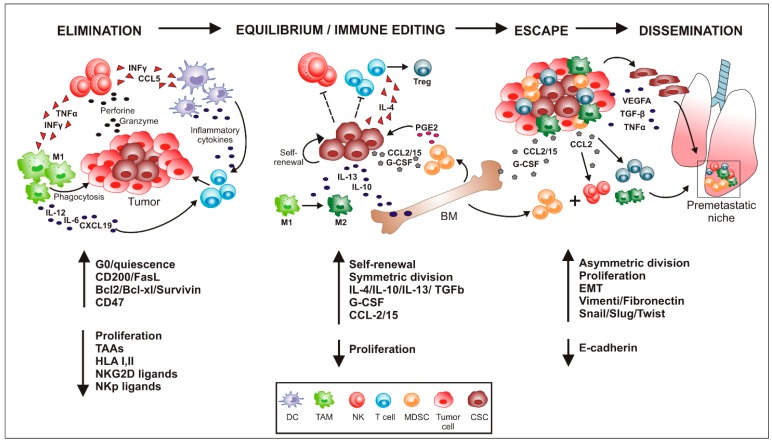
Cross-talks between cancer stem cells and innate immunity cellular components in the different phases of neoplastic progression. Schematic representation of the cross-talks between immune cells and cancer stem cells (CSCs). In the elimination phase, the different components of the innate immunity attack tumor mass but fail to eradicate CSCs, thanks to their ability to enter quiescence, activate anti-apoptotic pathways (overexpression of Bcl2/Bcl-x and Survivin), “don’t eat me” signal (CD47), and down-regulate the expression of tumor-associated antigens (TAAs), human leukocyte antigen (HLA) as well as ligands for natural killer cell (NK)-mediated recognition. Once survived this step, CSCs propagate themselves (self-renewal) and edit the immune system through the release of cytokines and soluble factors (IL-4, IL-13, G-CSF and CCL2/15) that activate the bone marrow (BM) towards an emergency myelopoiesis, characterized by expansion of myeloid suppressor cells. Thus, CSCs create a ‘permissive immunosuppressive tumor microenvironment’ (escape phase) that permits them to proliferate and differentiate, leading to tumor relapse. As the tumor grows, some CSCs lose their epithelial phenotype (reduced expression of E-cadherin) and acquire mesenchymal properties (i.e., increased expression of Vimentin, Fibronectin, Snail, Slug and Twist), through the epithelial to mesenchymal transition process (EMT), and leave the tumor site to enter the blood stream. In the meantime, the ongoing cancer-related inflammation produces cytokines and chemokines that promote accumulation of myeloid-derived suppressor cell (MDSCs), Treg, NKs and tumor-associated macrophages (TAMs) in distant tissues (i.e., lungs) to create pre-metastatic niches, where circulating CSCs will home and generate metastatic foci. (DC, Dendritic cell).

It has been demonstrated that the rare tumor cells able to survive the elimination phase are mostly cancer stem cells (CSCs) [10]. Even if their origin is not yet clear, the more trusted theory defines CSCs as normal stem cells that have accumulated neoplastic mutations. Due to their ability to develop into various cell types and support tissue regeneration, stem cells simultaneously became the “holy grail” of regenerative medicine, and the “evil contender” of anticancer therapy. Indeed, CSCs are considered responsible of tumor outgrowth, maintenance and progression, as well as resistance to anticancer treatments [11]. Thanks to their ability to enter quiescence and to express multidrug resistance extrusion pumps, CSCs survive conventional therapies (i.e., chemo and radio therapy) and orchestrate the metastatic spread to distant tissues. Identified for the first time in 1997 by Dick and Bonnet in leukemia [12], to date, CSCs have been described in almost all neoplastic tissues. Even if a universal marker for their identification is lacking, according to the tissue of origin, CSCs can be isolated on the base of the expression of specific surface markers, such as CD133, ALDH, c-kit [13] and CD44/CD24, as well as stemness-associated master gene regulators (e.g., Nanog, Sox2 and Oct4). In addition, CSCs are characterized by the capability to perpetuate themselves (self-renewal) and/or differentiate into all the different cell subsets of the originating tissue, together with the ability to grow in vitro as rounded structures called spheroids, resembling the 3D structure of the tumor mass [14]. Thanks to these double skills, CSCs maintain a stem reservoir and, simultaneously, sustain tumor growth. The balance between self-renewal and differentiation is controlled by the niche, a well-defined location where CSCs reside. Through the activation of specific pathways (i.e., Notch, Wnt/ß catenin and Sonic Hedgehog), the niche regulates the proliferative status of CSCs in response to cell-to-cell contacts and soluble factors released from other components of the TME. Accumulating evidences indicate that the interplay between innate immune components and CSCs in the different phases of cancer development acts as a key determinant of TME (Figure 1).

## 2. Elimination of Tumor Bulk

Microbial infections, autoimmunity and immune deregulation may instate chronic inflammatory conditions, which determine increased susceptibility to cancer development [2,15]. According to this causal association, obesity-associated inflammation and inflammaging are characterized by an increased risk of tumor development [16]. Metabolic and inflammatory disorders typical of obese people correlate with body–mass index (BMI) and increased DNA lesions [17], as well as with cancer growth, by favoring cancer cell proliferation, migration and resistance to apoptosis [18]. Further, the inflammatory connection postulated between aging and cancer appears to support changes in chromatin functions, reductions in autophagy capacity, and dysbiosis, which concur to create a pro-tumorigenic environment [18].

A major challenge is to understand how these conditions predisposing to tumor development intersect with the immune and inflammatory responses, and how the nascent tumor reprograms the immune system, gradually disarming its anti-tumor potential.

During the early stages of tumor development (elimination phase), immune cells retain their immunosurveillance properties, and anti-tumor immunity prevails. In cooperation with adaptive immunity (T and B lymphocytes), innate immune cells (macrophages, neutrophils, DCs and NK) provide a robust first line of defense against cancer cells, detecting and eliminating the more immunogenic cancer cells and counteracting spontaneous tumor growth [9,19]. NK cells possess potent cytolytic activity, to rapidly kill virally-infected or malignantly transformed cells in a manner unrestricted to the major histocompatibility complex class I (MHC-I), and secrete various effector cytokines and chemokines, like IFNγ, tumor necrosis factor alpha (TNFα), GM-CSF, CCL5 and IL-22, involved in the recruitment and activation of DCs and TAMs [20,21]. The activation of NK cells relies on the integration of signals derived from an array of cell surfaces activating (CD16 and NKG2D), natural cytotoxicity-mediating receptors (NKp30, NKp44, and NKp46) and inhibitory receptors (KIR, CD94/NKG2A and CD300a) [22,23,24].

TME-targeted, NK-mediated responses are triggered via: i) binding of the NKG2D to its ligands (stress-inducible MHC-Ib molecules, DNA damage responses); ii) binding of NKp30 and NKp44 with their respective ligands; and iii) antibody-dependent cellular cytotoxicity (ADCC), where the CD16 Fc receptor on NK cells binds to cell-bound antibodies [25,26]. In all cases, upon conjugations with target cells, NK cells release cytotoxic and proteolytic granules containing perforin and granzymes, for target cell lysis [27], and/or inducing target cell apoptosis via TNFα, Fas ligand (FasL), and TNF-related apoptosis-inducing ligand (TRAIL) [28,29].

In addition to their direct role in tumor surveillance, NK cells contribute to the formation of an efficient anti-tumor microenvironment through the early and rapid production of anti-tumor effector cytokines, such as IFNγ, which act in a paracrine manner on the other immune components of the TME. IFNγ reprograms TAMs into a tumoricidal (M1) phenotype [30,31], and promotes anti-tumor T-cell responses, inducing tumor-specific T cell memory [32], facilitating the differentiation of Th1 cells, and inhibiting the expression of exhaustion marker PD-1 on CD8^+^T cells [33]. Additionally, the cytokine-mediated (i.e., IFNγ and TNFα) interplay between NK cells, effector T cells and TAMs boosts differentiation of cytotoxic T cells (CTLs), enhances macrophage phagocytosis and the cytotoxic ability of NK cells, and increases the recruitment of cytotoxic TANs [34,35]. Moreover, in several mouse models, it has been shown that NK cells are required for the accumulation of conventional type I dendritic cells (cDC1) in TME. In fact, cDC1 are recalled through the secretion of CCL5 and XCL1 chemoattractants, resulting in increased priming and activation of anti-tumor T cells, and stimulating the overall effector immune response [36]. DCs represent not only the most important antigen-presenting cells (APCs), but also the link between innate and adaptive immunity, playing a key role in the priming and consolidation of anti-tumor adaptive immune response [37]. The ingested tumor-derived material is processed into peptides by immature DCs, and presented to T lymphocytes on MHC-I or MHC-II molecules. To efficiently drive the clonal proliferation and activation of CD4^+^ T helper (Th) or CD8^+^ CTL cells, DCs need to express co-stimulatory molecules, such as CD80, CD86, CD40, OX40L, or ICOSL, and produce stimulatory inflammatory cytokines (i.e., IL-6, IL-12, IL-23 and TNFα). In turn, DCs and T helper cells support the activation of NK, NKT and macrophages [38,39].

Activated macrophages can exhibit both proinflammatory (“M1 type”, driven by lipopolysaccharide (LPS) and IFNγ) or anti-inflammatory (“M2 type”, driven by IL-4 and IL-13) phenotype. The macrophage polarization status can be dynamically reprogramed during tumor development [40]. In the early steps of tumorigenesis, TAMs display M1 polarization state and play a relevant role in the elimination of immunogenic cancer cells, and in restraining tumor growth, through the expression of high levels of reactive nitrogen and oxygen intermediates and the secretion of pro-inflammatory and immune-stimulatory cytokines, such as IL-12, IL-6, IL-1, IL-23, CXCL19 and CXCL10. Then, these cytokines stimulate the development of Th1, Th17, NK and NKT cells [41,42]. In addition, macrophages could contribute to the elimination of nascent tumor cells through the release of nitric oxide (NO) and TNFα, which can be induced through NK2GD triggering [43]. Other stimuli, such as IFNγ, and anti-CD40 and toll-like receptor (TLR) ligands, can induce the release of NO and TNFα, thereby stimulating tumoricidal activity of macrophages [44]. Thus, a high M1/M2 ratio is predominantly associated with improved survival in multiple cancers [45,46].

**Figure 2:**
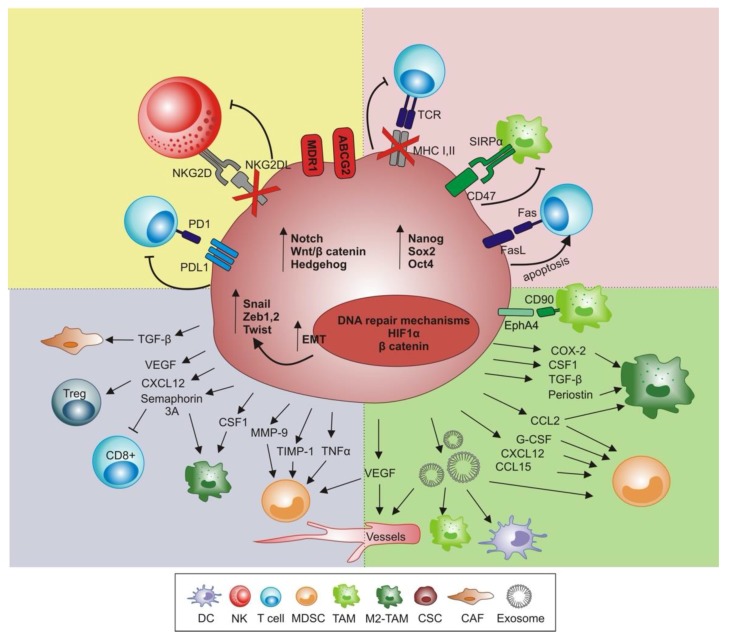
Mechanisms of CSCs immune escape. The figure summarizes the CSC features and CSCs-mediated immunosuppressive events during neoplastic progression. It must be taken into account that while the precise kinetics of the immunosuppressive events utilized by CSCs remains doubtful, it is possible to postulate that the activation of part of these mechanisms occurs in the very early stages of tumor development, when instead the non-CSCs are effectively eliminated by the immune response. These immunosuppressive mechanisms are likely to remain active, allowing the disease recurrence. CSCs overexpress PD1 ligand (PDL1) or down-regulate NKG2D ligand (NKG2DL) to respectively inhibit T and NK cells’ antitumor functions. To maintain a low immunogenic profile, CSCs down-regulate the expression of MHC class I and II, thus impairing TCR-mediated recognition, and express the “don’t eat me” signal (CD47), which inhibits the phagocytic activity of SIRP1α^+^ macrophages. At the same time, expression of Fas ligand (Fas L) by CSCs induces apoptosis of effector T cells. In addition, CSCs express multi drug resistant pumps (e.g., MDR1 and ABCG2) and activate pathways (e.g., Notch, Wnt/β-catenin and Sonic Hedgehog) to survive anti-neoplastic treatments and enter quiescence. In support of tumor growth, CSCs gradually create a tumor-promoting environment, by releasing cytokines and soluble factors that affect the polarization of TAMs towards an M2 protumor phenotype, and favoring expansion of myeloid-derived suppressor cells (MDSCs). In addition, CSCs release exosomes endowed with several effects, according to their cargo. Exosomes impair dendritic cell (DC) maturation, modulate the inflammatory response of tumor-associated macrophages (TAMs) and promote neoangiogenesis. The formation of new vessels is fostered also by vascular endothelial growth factor (VEGF), released directly by CSCs, that in concert with other cytokines and soluble factors orchestrates the creation of a prone premetastatic niche. At the same time, Treg are recruited into the premetastatic niche, and the cancer associated fibroblasts (CAFs) are functionally shaped to remodel extracellular matrix and to sustain CSC migration and tumor progression. During this last phase, CSCs respond to the increased HIF-1α levels, activating epithelial to mesenchymal transition (EMT) leading to the acquisition of mesenchymal features (e.g., expression of Snail. Zeb1/2, and Twist).

Similar to the M1/M2 phenotype of TAMs, it has been proposed that tumor-associated neutrophils (TANs) can be polarized toward anti-tumor N1 or pro-tumorigenic N2 phenotype. During the first steps of tumorigenesis, the recruitment of TANs to the TME is mediated by CXCR2 ligands, such as CXCL1, CXCL2 and CXCL5, secreted by cancer and stromal cells. Once within TME, the presence of type-1 IFNs shapes their phenotype towards an anti-tumor N1 polarization state, associated with increased tumor cytotoxicity, high neutrophil extracellular traps (NETs) expression, and enhanced TNFα and reactive oxygen intermediates expression [47,48]. However, despite the fact that during the early phase of cancer development a vigorous immune attack contributes to the elimination of cancer cells, simultaneously it also selects the less immunogenic variants, invisible to immune detection, such as the CSCs. The low immunogenicity features and the quiescent behavior exhibited by CSCs, which allow their immune escape, are described below (see also Figure 2).

### 2.1. Equilibrium and Survival of CSCs

The ability of poorly immunogenic CSCs to persist in a dormant state provides a rationale for both their immuno-evasion and resistance to anticancer therapies. In fact, in response to different signals, CSCs can exit the cell cycle and enter quiescence, a G0 cell cycle arrest status that is mainly controlled by the transcription factor homeobox protein nanog (NANOG) through the Wnt/β catenin pathway [49]. Furthermore, compared to bulk tumor cells, CSCs exhibit increased DNA repair and reduced apoptosis [50,51], along with enhanced expression of drug efflux pumps, such as ABCG2 and MDR1, which makes them inherently drug resistant [52] and strengthens their stemness properties [53,54].

CSCs exhibit various properties to directly evade effector mechanisms of immune cells (Figure 2). Among all, CSCs down-regulate the expression of MHC class I and II molecules. A comparison study between CSCs, grown as spheroids, and non-stem cancer cells (non-CSCs) isolated from glioblastoma [55] and melanoma [56], demonstrated that CSCs are characterized by weak or no expression of MHC class I molecules, suggesting an impaired recognition by CD8^+^ T cells. In agreement, phenotypic analysis of CSCs, isolated from primary colorectal carcinoma specimens and co-cultured with peripheral blood mononuclear cell (PBMC), demonstrated their weak immunogenicity, paralleled by low expression of class I and II human leukocyte antigen (HLA) and high levels of immunomodulating cytokines, such as IL-4 [57].

In a study on tumor relapse in head and neck squamous cell carcinoma, it has been demonstrated that first line radiotherapy treatment is associated with an enrichment of tumor cells characterized by defective antigen processing machinery (APM) and low expression of HLA class I molecule [58], that correlated with a reduction in the patient’s overall survival. Down-regulated or defective APM (i.e., transporter associated antigen processing proteins TAP or ß-microglobulin) has been also demonstrated in CSCs [59], and correlated with the absence or suboptimal expression of tumor-associated antigens (TAAs) [60]. In line with this, reduced expression of HLA I or TAP molecules was reported in CSCs isolated from melanoma [56], lung cancer [61], head and neck squamous cell carcinoma (HNSCC) [58] and colorectal cancer [57], in comparison to the non-CSCs counterparts. 

Within the framework of an extreme capacity for immune-evasion and survival, high levels of CD200, FasL, and anti-apoptotic proteins like Bcl-2, Bcl-xL, or Survivin, were shown to protect CSCs against chemotherapeutic drugs, and increase resistance toward apoptosis-inducing immune effectors like T or NK cells [62,63].

More recently, a link between death receptor CD95/Fas, type I interferons (IFN-I)-dependent activation of STAT1, and stemness, has been described in different cancer types [64]. In fact, chronic stimulation of CD95 in tumor cells has been reported to activate the IFN-I pathway and result in increased expression of stem-like markers in breast cancer, glioblastoma and squamous carcinoma [65]. CSCs are also able to convert a subset of immature DCs into TGF-β-secreted cells, thus driving expansion of regulatory T cells (Treg) in lymphoid organs [66].

Next to resisting T cell-mediated eradication, CSCs also escape NK anti-tumor activity. Several works demonstrated that CSCs down-regulate NK-activating NKG2D ligands, thus impairing their innate immune response against tumors [55,67]. Additionally, CSCs express low levels of ligands for the NK cell activator receptor NKp44, and no ligands for NKp30, NKp46 and CD16, while increasing inhibitory NK cell receptors ligands [10,68]. Thus, as demonstrated by Reim et al., CD44^high^/CD24^low^ breast CSCs selectively escape from NK cell-mediated killing and Trastuzumab-dependent ADCC [69]. Moreover, in several types of malignancies, including lung, pancreatic and hepatocellular carcinoma, CSCs, compared to non-CSCs, were reported to express increased levels of the “don’t eat me” signal CD47, thus preventing their phagocytosis by signal regulatory protein α (SIRPα) on TAMs [70,71,72]. In line with this, studies showed an increased phagocytosis of CSCs by macrophages upon blocking of CD47 [70,73].

However, some reports showed similar HLA expression or higher new antigen expression in CSCs, as compared to the non-CSCs counterpart, suggesting that CSCs also present immunogenic potential, able to activate T-cell mediated anti-tumor responses [74]. A study on colon cancer showed no differences in HLA class I expression between CSCs and non-CSCs [75]. Similarly, Chikamatsu et al. did not identify differences in HLA class I and TAP expression in CSCs vs non-CSCs in head and neck squamous cell carcinoma (HNSCC), although CSCs displayed reduced levels of TAP2. Conflicting results were obtained on NK-mediated recognition and elimination of CSCs [76]. By using several cancer cell lines and primary samples, Ames et al. demonstrated that the CSCs (identified as CD24^+^/CD44^+^/CD133^+^/ALDH^bright^) present higher levels of MICA/MICB, Fas and DR5, NK activator ligands, as compared to non-CSCs [77].

The contrasting evidences present in the literature could be partially due to lack of universal markers for CSCs identification in the different tissues, and different non-standardized techniques used for their isolation and culture [74].

### 2.2. CSCs Awakening and Immunoediting

CSCs survive tumor eradication in a state of tumor dormancy, without losing their malignant potential, by orchestrating the shift from a state of immunosurveillance/equilibrium to an escape phase (Figure 2).

The lack of favorable microenvironmental conditions and specific stimuli for cell proliferation maintain CSCs in quiescence, preventing the formation of a tumor mass [60]. In primary samples of acute myeloid leukemia, Shapiro’s group demonstrated that leukemia cells collected at tumor relapse had undergone a lower number of divisions, compare to cells collected at diagnosis, and presented features resembling the stem cell subset [78]. These results indicate that tumor growth is triggered by an aggressive and slowly dividing cancer cell clone, the CSCs, endowed with tolerogenic properties.

During the escape phase, CSCs secrete cytokines, chemokines and soluble factors that activate different pathways, aiming at tipping the balance towards immune tolerance, in order to suppress and edit the immune system and create a pro-tumoral niche [79]. In concert with Treg, TAMs and MDSCs represent the major orchestrators of this process, as they boost an immune-tolerant TME through the secretion of immunosuppressive molecules, such as IL-10, TGF-β and prostaglandins [80]. In addition, they inhibit the secretion of IL-12 by DCs, blocking effective Th1 response and excluding NK, NKT and effector T cells [81,82,83,84]. The immunosuppressive evolution of TME, driven by immunosuppressive factors and cytokines (i.e., TGF-β ), subsequently induces a population of pro-angiogenic, N2-polarized TANs [47].

TAMs and MDSCs abundantly expand in tumor bearers as a result of an “emergency myelopoiesis”, which leads to pathological expansion of tumor-promoting myeloid cells, endowed with tumor-promoting activities [85,86]. Similarly, with infections [87,88], emergency myelopoiesis occurring in cancer is an attempt to ensure proper supply of immune cells for the increased demand [86]. Nevertheless, in addition to the local editing of immune cells occurring in the TME, cancers deliver systemic immunological stresses that alter the differentiation of bone marrow progenitors, hence affecting magnitude, composition and specialized functions of the hematopoietic output [89,90].

Subsequently, tumor-infiltrating myeloid cells, mobilized from the bone marrow to the periphery, are recruited to the tumor site, where they encounter extreme microphysiological conditions (i.e., hypoxia, low glucose levels, low pH and inflammatory signals) that further boost their reprograming towards a tumor-promoting phenotype [84,91]. CSCs participate in shaping myeloid cell phenotype by releasing inflammatory mediators, such as IL-10 and IL-13, that promote the differentiation of immature myeloid cells [74]. In addition, CSCs-derived G-CSF, CCL2, CCL15 and CXCL12 recruit MDSCs into the TME [92,93]. In a mouse model of pancreatic cancer (PC), it has been demonstrated that monocytic-MDSCs (M-MDSCs), identified as CD11b^+^/Gr1^+^/Ly6G^-^/Ly6C^high^, stimulate the expansion of ALDH^brigh^ CSCs, and increase the expression of genes associated with the EMT process [94]. Similar results were obtained analyzing primary samples of M-MDSCs isolated from peripheral blood of PC-bearing patients. M-MDSCs increase the percentage of CSCs and promote acquisition of mesenchymal features. Interestingly, the authors proved that the CSCs stimulation was mediated by the activation of the STAT3 pathway, and that the blockade of STAT3 activation restored the basal levels of ALDH^brigh^ CSCs [94]. Wang et al. reported also that granulocytic-MDSCs (G-MDSCs) enhance colorectal cancer (CRC) cell stemness via exosomes and exosomal S100A9 in the tumor microenvironment, especially under hypoxic conditions. In line with this, plasma exosomal S100A9 in CRC patients is markedly higher than that in healthy subjects [95].

It was also reported that MDSCs induce the expression of microRNA101 in ovarian cancer cells, which subsequently repress the corepressor gene C-terminal binding protein-2 (CtBP2), increasing their stemness and tumorigenic potential [96]. Additionally, prostaglandin E2 (PGE2), secreted by MDSCs, boosted the stemness of cervical cancer cells [97], while MDSCs enhanced stemness-properties of breast tumor cells through the secretion of IL-6 and nitric oxide (NO), in a STAT3-dependent manner [98]. In line with this, the STAT3/IL-6 pathway was reported to be involved in the increased frequency of CD44^+^ hepatocellular carcinoma (HCC) stem cells, when co-cultured with TAMs isolated from HCC-bearing patients. As demonstrated by Wan and colleagues, disruption of this axis, through STAT3 or IL-6 inhibitors, resulted in the impairment of the CSCs’ expansion, both in vivo and in vitro [99]. These results support the idea that TAMs contribute to neoplastic progression also by nurturing the cancer stem cell niche [100,101].

An existing mutual crosstalk indicates that TAMs release cytokines that contribute to the activation of CSCs, which in turn release factors contributing to switching TAMs toward an M2-polarized and tumor-promoting phenotype [81]. In this scenario, periostin, a secreted extracellular matrix protein produced by glioma stem cells, was shown to attract M2 TAMs, hence promoting tumor growth [102]. Colony-stimulating factor 1 (CSF-1) and CCL2, enriched in CSC niches, mediated the recruitment of TAMs and shifted the polarization of the monocyte pool toward M2-activation state [103]. As demonstrated in ovarian cancer [104] and glioma [105], CSCs release cycloxygenase2 and CCL2, as well as CSFs and TGF-β, to orchestrate the polarization of TAMs [106].

Interestingly, pancreatic ductal adenocarcinoma (PDAC) cells polarize TAMs toward an M2 phenotype, which in turn actively secrete high levels of ISG15, an interferon-stimulated gene which promotes self-renewal, tumorigenic, chemoresistant and migratory capacities of PDAC CSCs in vivo and in vitro [107,108].

Reciprocally, TAMs play a key role in maintaining the CSCs’ functions [109]. In non-small-cell lung cancer (NSCLC) and HCC, IL-6 produced by TAMs supported the expansion and drug resistance of CSCs in a STA3-dependent manner [99,100]. In addition, M2 TAMs promoted invasion and proliferation of CSCs by the secretion of TGF-β in HCC, along with the release of PDGF, IL-8 and CXCL12 [110,111]. VEGF, which is produced not only by M2 TAMs but also by CSCs themselves, promotes angiogenesis, tumorigenicity and stem-like phenotypes of CSC themselves [112,113]. TAM-initiated Wnt signaling activation, during CSCs/TAM interaction, constitutes a positive feedback loop that contributes to the maintenance of stemness of ovarian CSCs [114].

Besides the paracrine interaction via soluble molecules, TAMs interact with CSCs also in a cell–cell contact dependent manner; in particular, the ligation of CD90 to Ephrin type-A receptor 4 (EphA4), expressed on the surface of CSCs, induces the production of IL-6, IL-18 and GM-CSF, which in turn sustain stem cell-like niche [115].

Additionally, much evidence in the literature highlights the CSCs-exosome role in creating a permissive pro-tumoral microenvironment. In this regard, it was reported that glioblastoma stem cell-derived exosomes skew monocytes toward an immunosuppressive M2 phenotype, through the STAT3 pathway, fostering PD-L1 expression and cytokines production, such as MCP-3 and CXCl1, which further enhance the recruitment of myeloid cells into TME. [116]. Similarly, Domenis et al. showed that peripheral blood monocytes, from healthy donors, exposed to glioma stem cell-derived exosomes had increased IL-10 and Arg1, and downregulated class II HLA (HLA-DR) expression, thus inducing cells with characteristics similar to M-MDSC [117]. In line with this, TGF-β, as well as C1q- and semaphorins-containing exosomes secreted by human and mouse tumor-educated mesenchymal stem cells, drive accelerated breast-cancer progression by inducing differentiation of M-MDSCs into immunosuppressive PDL-1^+^CD206^+^Arg1^+^IL-10^+^ M2-polarized macrophages [118]. In a renal cancer model, CSCs impaired DCs maturation and T cell immune response, fostering tumor immune-escape, through the release of HLA-G-carrying extracellular vesicles [119]. Moreover, mesenchymal stem cells-derived exosomes contribute to modulating the inflammatory responses of TAMs, inducing higher expression of anti-inflammatory IL-10 and TGF-β transcripts and reducing levels of pro-inflammatory IL-1β, IL-6, TNFα and IL12p40 [120].

Colorectal cancer stem cell-derived exosomes prolonged the survival of bone marrow-derived neutrophils, and induced a pro-tumoral phenotype in TANs, by increasing IL-1β expression [121].

In rat models of HCC, hepatic CSCs-derived exosomes displayed protumor functions, influencing apoptosis, angiogenesis, metastasis and invasiveness, as well as EMT of tumor cells, via altering the expression of targeted molecules, such as p53, Bcl2, VEGF, TGF-β and matrix metalloproteinase (MMP)-9 [122]. Sun et al. recently reported that glioma stem cell-derived exosomes promoted angiogenesis by containing high levels of miRNA-21, which in turn up-regulates VEGF [123]. The extracellular CSC-derived vesicles in human renal cell carcinoma have been shown to contain several proangiogenic mRNAs, such as VEGF, angiopoietin1, MMP-2 and MMP-9, thereby modifying the TME by triggering angiogenesis and promoting the formation of premetastatic niches [124].

In line with this, CSC-derived exosomes induced metastasis by promoting EMT in renal cell carcinoma [125] and thyroid cancer, via the transfer of miRNA-19b-3p and non-coding-RNAs, respectively [126].

### 2.3. Dissemination and Metastases

The last phase of the neoplastic progression is tumor metastatization, the spread of tumor cells from the primary site to non-adjacent tissues, where they give rise to a tumor mass resembling the primary tumor. Metastases is the major cause of cancer-related death, accounting for almost 90% of the cases. During the metastatization process, tumor cells acquire the capability to degrade the extracellular matrix, leave the tumor site, penetrate into the blood vessels (intravasation) and exit in distant tissues (extravasation), where they conclude the metastatic colonization. Strikingly, primary tumor-derived factors actively condition the nutrients, extracellular matrix and immune cell environment of a distant organ, to make it accessible to metastatic seeding [127]. Thus, the pre-metastatic niche can be primed and established through a dynamic interplay among tumor-derived factors, tumor-mobilized bone marrow-derived cells, and local stromal components [128].

Stephen Paget in 1889 proposed the “seeds and soil” theory, according to which metastatization occurs only when designated tumor cells reach specific tissues already activated to promote tumor cell growth and survival [129]. To date, it is clear that CSCs are the designated tumor cells, since they possess the capability to survive sufficiently in the blood stream, and subsequently to colonize distant tissues. Here, CSCs can remain dormant for a long time, to then restart proliferation and differentiation, thus recapitulating the primary tumor phenotype. To acquire migratory ability, CSCs undergo the EMT [130]. EMT is a program that in non-pathological tissues occurs during embryonic developments; in neoplastic tissues, instead, it sustains tumor cell dissemination to distant organs [131]. During EMT, CSCs lose their polarity and cell-to-cell junctions, acquiring a spindle shape and mesenchymal features, such as motility and invasiveness. Indeed, hallmarks of EMT are down-regulations of epithelial markers (i.e., E-cadherin) and up-regulations of mesenchymal ones (i.e., Vimentin, Fibronectin and V-cadherin) [132].

In the tumor context, activation of EMT can be triggered by several factors (i.e., hypoxia, nutrient deprivation and starvation), and pathways including NF-κB, Notch, and Wnt/β catenin, as well as TGF-β. Indeed, different from the stage of tumor onset, in the later stage of neoplastic progression, TGF-β orchestrates CSCs’ metastatic spread through the activation of Smad proteins, which in turn regulate the expression of target genes, such as master transcription factors Snail, Zeb1, Zeb2 and Twist, that drive the EMT program [133,134,135].

CSCs can directly produce TGF-β, which acts in an autocrine manner, to sustain their own metastatic potential, or affect adjacent/distant cells [136]. Tumor-derived TGF-β, together with other factors, such as VEGFA and TNFα, stimulates the release of chemoattractants, such as S100A8 and S100A9, that mediate the recruitment of immune cells in the pre-metastatic niche [137]. Indeed, as foreseen by Paget [129], in the metastatic process a prone niche is fundamental for tumor cell seeding and tissue colonization. In accordance with this, Sceneay J et al. demonstrated that primary tumor hypoxia induces tumor cells to release CCL2, G-MCSF, TNFα, VEGF, TIMP-1 and MMP-9, which orchestrate the recruitment of MDSCs (CD11b^+^/Ly6C^med^/Ly6G^+^) in the pre-metastatic lung niche [138]. Among all these, tumor-derived CCL2 stimulates the accumulation of myeloid cells and controls the differentiation of MDSCs into immunosuppressive macrophages, which in turn enhance metastases formation (e.g., CSCs establishment, proliferation and differentiation). In addition, hypoxia-induced tumor-released factors recall Treg and NK cells in the premetastatic niche [139]. However, once in situ, NK cells present an impaired differentiation status and reduced cytotoxic potential [138], thus creating an immunosuppressive environment favoring the settling down of CSCs and the growth of metastases [140]. Preclinical evidence in both Lewis lung cancer (LLC) and melanoma (B16) mouse models [141] reported that VEGFR1-expressing, bone marrow-derived hematopoietic progenitors accumulate in the pre-metastatic sites, before the detection of metastatic cells.

Moreover, additional immune cells are involved in the promotion of the metastatic process. TAMs, for instance, regulate stroma invasion and intravasation of tumor cells. In response to CSF1 (secreted by tumor cells), TAMs release the epidermal growth factor (EFG) that binds its receptor on the surface of tumor cells, activating signaling of motility, matrix degradation and invasion of the surrounding tissue [142]. In addition, TAMs secrete CCL18 [143] and SPARC [144], which enhance the adhesive capability of tumor cells to the extracellular matrix, helping CSCs to enter blood vessels and migrate to the metastatic niche. On the other side, tumor-released VEGFA, semaphorin 3A and CXCL12 recruit TAMs in pre metastatic niches, where they suppress the cytotoxic activity of CD8+ T cells and promote recruitment of Treg cells [145]. DCs, as well, participate to the creation of an immunosuppresive niche, inducing the expansion of Treg and the inhibition of CD8+ T cells, as demonstrated in liver metastasis from pancreatic cancer cells [146].

De Visser and colleagues, instead, recently demonstrated in a model of mouse breast cancer that neutrophils create a prone metastatic niche for tumor circulating cells. The authors showed that neutrophils release nitric oxide (NO) to suppress CD8^+^ T cells, allowing the growth of metastatic foci. Neutrophil depletion, using anti-Ly6G antibody, did not affect primary tumor growth, but was associated with a reduction in lung and lymph node metastasis [147]. More recently, the same authors demonstrated that a mechanistic link between the loss of p53 in cancer cells, secretion of Wnt ligands and systemic neutrophilia potentiates metastatic progression [148]. Further, in support of an active role of neutrophils in cancer cells spreading, Wculek et al. proved that neutrophil-derived leukotrienes orchestrate lung metastatization of breast cancer, by enhancing the proliferation of high tumorigenic CSCs [149].

Some evidence leads to speculation that soluble factors, exosomes and extracellular vesicles, not only from CSCs but also from the microenvironment of the primary tumor, could promote, at least in part, the construction of premetastatic niches, by modulating the differentiation of the cellular components of TME [150,151]. It is known that CSCs modulate stromal activity; for example, transforming fibroblasts into cancer-associated fibroblasts (CAFs) through cytokine secretion (i.e., TGF-β) [152]. As a consequence, CAFs release other cytokines, chemokines, growth factors and matrix metalloproteinases (i.e., TGF-β, PGE2, CXCL12, VEGF, MMPs, etc.) that sustain tumor progression, EMT transition and cancer stemness [153,154,155]. In turn, CAFs-derived exosomes contribute to the conversion of cancer cells into CSCs, for example, via activation of Wnt and NOTCH signaling pathways, and support the self-renewal and stemness properties of existing CSCs [156,157]. Recently, Su S. et al. demonstrated that a subset of CD10- and GPR77-expressing CAFs promote tumor formation and chemoresistance, by favoring the formation of a CSCs niche [158]. Accordingly, Donnarumma et al. identified three miRNAs (miR-21, -378e and -143), enriched in exosomes derived from CAFs, which could significantly increase the ability to promote stemness and EMT phenotype of breast cancer cells [159]. Furthermore, CAFs secreted exosomes fostering breast cancer cell migration through Wnt-planar cell polarity (PCP) signaling [160]. Such interactions may create a niche for tumor growth and metastasis [161]. As the niche is established, CSCs recruit TAMs, CAFs and other stromal cells, to establish the paracrine loops that supply CSCs with TNFα and TGF-β for CSC maintenance [111,140,162]. In the meantime, the surrounding CAFs secrete MMPs and cathepsins to further break down the extracellular matrix, which in turn releases TGF-β and various growth factors like VEGF-A, hence allowing tumor expansion [163].

## 3. Immunological Targeting of CSCs

As discussed, CSCs are able to shape the TME by attracting immunosuppressive innate cell subsets and inhibiting effector T cells. On the other hand, stromal cells and infiltrating immune cells support CSCs’ self-renewal, tumorigenicity and metastasis [74]. Additionally, CSCs resist conventional cancer therapies, primarily due to their high capability to repair DNA damage and to proliferate slowly [11,105,164]. Radiation is commonly used to treat many types of cancer, and its combination with immunotherapy is considered promising [165]. This combination is expected to have synergistic effects, stemming from both local and systemic tumor control, due to the unique and intriguing interactions between radiation and the immune system [166]. Interestingly, radiotherapy induces pro-inflammatory cytokines in TME (i.e., IL-1β, TNFα, TGF-β, CXCL12, IL-6, MMPs, etc.) [167], which lead to the up-regulation of reactive oxygen species (ROS) scavengers in CSCs and the activation of downstream STAT3 signaling, with development of highly invasive CSCs phenotypes [168,169]. These results highlight the need for new strategies that may selectively target the CSCs, highlighting their therapeutic potential combined with conventional antineoplastic therapy [170].

Combination immunotherapies would be an ideal approach to restore anti-tumor immunity against CSCs. As mentioned, active STAT3 signaling plays an important role in CSCs/immune cells interaction, including the generation of M2 TAMs, MDSCs, and the effect of IL-6 and IL-17 on the stemness and suppressive (e.g., PD-L1 expression) properties of CSCs [94,99,116]. Many of these effects could be reversed by inhibition of STAT3, rendering this molecule an attractive therapeutic target to block CSCs-associated tumor immune evasion [171]. For example, inhibitor OPB-51602 is showing promising effects against non-small cell lung carcinoma [172], and the STAT3 inhibitor napabucasin was shown to reduce both expression of stemness-related genes and sphere formation by glioblastoma cells [173].

Furthermore, the SIRPα ligand CD47 is overexpressed by CSCs, and could represents another possible target for therapy [70]. Several studies showed an increased phagocytosis of CSCs by macrophages upon blocking of CD47, and multiple CD47 inhibitors are being tested in ongoing clinical trials [73,174].

Additionally, CSCs were shown to express increased levels of the immune checkpoint PD-L1, which correlated with EMT [175]. In turn, in gastric cancer, PD-L1 binding to PD-1 enhanced CSCs proliferation [176]. Gupta et al. reported that PD-L1 knock-down in B16 murine melanoma cells significantly reduced the CSC population [177]. Together, these studies suggest that using immunotherapeutic agents, such as the checkpoint blockade inhibitors, may be an effective way of targeting CSCs.

TGF-β, secreted by Tregs, M2 macrophages or CSCs themselves, is a crucial mediator of immunosuppression that can be targeted by neutralizing antibodies [178]. The inhibition of the pro-angiogenic VEGF has also been proven beneficial as combination therapy in several tumors, and could be used to block the CSC-mediated angiogenesis [179].

Finally, CSCs might be eliminated by using specific immunotherapeutic approaches, such as drug-conjugated monoclonal antibodies, DC-based therapeutic vaccines, and chimeric antigen receptor- or T cell receptor-engineered T cells (CAR T-cells), targeting antigens that are characteristically expressed by CSCs [180,181,182,183,184]. While these approaches may help to design efficient therapies for eradication of CSCs, to this end it will be necessary to improve our knowledge of the biology of CSCs, and the cellular interactions they establish within the TME.

## 4. Conclusions

CSCs and immune cells are the key players in tumor onset, progression and metastasis formation, shaping the primary tumor microenvironment, as well as premetastatic sites, through triangular and reciprocal interactions with stroma cells. The biology of CSCs, and the complexity of their intricated microenvironmental cross-talk, have not yet been completely clarified. In particular, understanding of the mechanisms that guide the plasticity and phenotype of CSCs (i.e., immunogenicity, proliferation rate, differentiation, migration, etc.) during neoplastic evolution, as well as the identification of CSCs’ specific markers, represent the major limitations for CSC-targeted anticancer therapies, which aim at the complete eradication of the tumor and the effective prevention of relapses. Additional contributions to an effective therapeutic targeting of CSCs will likely come from a better understanding of how inflammatory circuits and immune cells sculpt CSCs functions, thus influencing their awakening and entry into the cell cycle, and promoting disease recurrence, as well as resistance and survival to anticancer therapies.

## Abbreviations

ADCC	antibody-dependent cellular cytotoxicity
APC	Antigen-presenting cell
APM	Antigen-processing machinery
BCL	B cell lymphoma
BMDC	Bone marrow-derived cell
CAF	Cancer-associated fibroblast
CCL	Chemokine (C-C motif) ligand
CSF1	Colony-stimulating factor 1
CXCL	Chemokine (C-X-C motif) ligand
CXCR	Chemokine (C-X-C motif) receptor
CRC	Colorectal cancer
CSC	Cancer stem cell
CTL	Cytotoxic T cell
DC	Dendritic cells
EGF	Epidermal growth factor
EMT	Epithelial-to-mesenchymal transition
GM-CSF	Granulocyte-macrophage colony-stimulating factor
G-MDSC	Granulocytic myeloid-derived suppressor cell
HCC	Hepatocellular carcinoma
HLA	Human leukocyte antigen
HNSCC	Head and neck squamous cell carcinoma
IFNγ	Interferon gamma
IL	Interleukin
iNOS	induced Nitric Oxide synthase
MDSC	Myeloid-derived suppressor cell
M-MDSC	Monocytic myeloid-derived suppressor cell
MHC	Major histocompatibility complex
NET	Neutrophil extracellular trap
NK	Natural Killer cells
NKG2D/A	Natural killer group 2 member D/A
NKT	Natural killer T cell
NO	Nitric oxide
NSCLC	Non-small cell lung cancer
PBMC	peripheral blood mononuclear cell
PC	Pancreatic cancer
PDAC	Pancreatic ductal adenocarcinoma
PGE2	Prostaglandin E2
SIRP	Signal regulatory protein
SPARC	Secreted protein acidic and rich in cysteine
STAT3	Signal transducer and activator of transcription 3
TAA	Tumor-associated antigen
TAM	Tumor-associated macrophages
TAN	Tumor-associated neutrophils
TGF	Transforming growth factor
Th	T helper cell
Treg	Regulatory T cell
TME	Tumor microenvironment
TNFα	Tumor necrosis factor alpha
TRAIL	TNF-related apoptosis-inducing ligand
VEGF	Vascular endothelial growth factor
XCL1	X-C Motif Chemokine Ligand 1

## References

[1] Hanahan D., Weinberg R.A. (2011). Hallmarks of cancer: The next generation. Cell.

[2] Mantovani A., Allavena P., Sica A., Balkwill F. (2008). Cancer-related inflammation. Nature.

[3] Balkwill F., Mantovani A. (2001). Inflammation and cancer: Back to virchow?. Lancet.

[4] Fridman W.H., Zitvogel L., Sautès-Fridman C., Kroemer G. (2017). The immune contexture in cancer prognosis and treatment. Nat. Rev. Clin. Oncol..

[5] Coussens L.M., Zitvogel L., Palucka A.K. (2013). Neutralizing tumor-promoting chronic inflammation: A magic bullet?. Science.

[6] De Visser K.E., Eichten A., Coussens L.M. (2006). Paradoxical roles of the immune system during cancer development. Nat. Rev. Cancer.

[7] Hinshaw D.C., Shevde L.A. (2019). The tumor microenvironment innately modulates cancer progression. Cancer Res..

[8] Dunn G.P., Old L.J., Schreiber R.D. (2004). The three es of cancer immunoediting. Annu. Rev. Immunol..

[9] Schreiber R.D., Old L.J., Smyth M.J. (2011). Cancer immunoediting: Integrating immunity’s roles in cancer suppression and promotion. Science.

[10] Bruttel V.S., Wischhusen J. (2014). Cancer stem cell immunology: Key to understanding tumorigenesis and tumor immune escape?. Front. Immunol..

[11] Batlle E., Clevers H. (2017). Cancer stem cells revisited. Nat. Med..

[12] Bonnet D., Dick J.E. (1997). Human acute myeloid leukemia is organized as a hierarchy that originates from a primitive hematopoietic cell. Nat. Med..

[13] Pastò A., Bellio C., Pilotto G., Ciminale V., Silic-Benussi M., Guzzo G., Rasola A., Frasson C., Nardo G., Zulato E. (2014). Cancer stem cells from epithelial ovarian cancer patients privilege oxidative phosphorylation, and resist glucose deprivation. Oncotarget.

[14] Sica A., Porta C., Amadori A., Pastò A. (2017). Tumor-associated myeloid cells as guiding forces of cancer cell stemness. Cancer Immunol. Immunother..

[15] Coussens L.M., Werb Z. (2002). Inflammation and cancer. Nature.

[16] Bottazzi B., Riboli E., Mantovani A. (2018). Aging, inflammation and cancer. Semin. Immunol..

[17] Włodarczyk M., Jabłonowska-Lietz B., Olejarz W., Nowicka G. (2018). Anthropometric and dietary factors as predictors of DNA damage in obese women. Nutrients.

[18] Włodarczyk M., Nowicka G. (2019). Obesity, DNA damage, and development of obesity-related diseases. Int. J. Mol. Sci..

[19] Teng M.W.L., Galon J., Fridman W.H., Smyth M.J. (2015). From mice to humans: Developments in cancer immunoediting. J. Clin. Invest..

[20] Castriconi R., Daga A., Dondero A., Zona G., Poliani P.L., Melotti A., Griffero F., Marubbi D., Spaziante R., Bellora F. (2009). NK cells recognize and kill human glioblastoma cells with stem cell-like properties. J. Immunol..

[21] Vitale M., Della Chiesa M., Carlomagno S., Pende D., Aricò M., Moretta L., Moretta A. (2005). NK-dependent DC maturation is mediated by TNFα and IFNγ released upon engagement of the NKp30 triggering receptor. Blood.

[22] Guia S., Fenis A., Vivier E., Narni-Mancinelli E. (2018). Activating and inhibitory receptors expressed on innate lymphoid cells. Semin. Immunopathol..

[23] Vivier E., Tomasello E., Baratin M., Walzer T., Ugolini S. (2008). Functions of natural killer cells. Nat. Immunol..

[24] Lanier L.L. (2001). On guard—Activating NK cell receptors. Nat. Immunol..

[25] Dambrauskas Z., Svensson H., Joshi M., Hyltander A., Naredi P., Iresjö B.M. (2014). Expression of major histocompatibility complex class I-related chain A/B (MICA/B) in pancreatic carcinoma. Int. J. Oncol..

[26] Gasser S., Orsulic S., Brown E.J., Raulet D.H. (2005). The DNA damage pathway regulates innate immune system ligands of the NKG2D receptor. Nature.

[27] Orange J.S. (2008). Formation and function of the lytic NK-cell immunological synapse. Nat. Rev. Immunol..

[28] Chyuan I.T., Tsai H.F., Liao H.J., Wu C.S., Hsu P.N. (2018). An apoptosis-independent role of TRAIL in suppressing joint inflammation and inhibiting T-cell activation in inflammatory arthritis. Cell. Mol. Immunol..

[29] Screpanti V., Wallin R.P.A., Ljunggren H.-G., Grandien A. (2001). A central role for death receptor-mediated apoptosis in the rejection of tumors by NK cells. J. Immunol..

[30] Kwon B. (2018). IFN-γ in tissue-immune homeostasis and antitumor immunity. Cell. Mol. Immunol..

[31] O’Sullivan T., Saddawi-Konefka R., Koebel W.V., Arthur C., White J.M., Uppaluri R., Andrews D.M., Ngiow S.F., Teng M.W.L., Smyth M.J. (2012). Cancer immunoediting by the innate immune system in the absence of adaptive immunity. J. Exp. Med..

[32] Kelly J.M., Darcy P.K., Markby J.L., Godfrey D.I., Takeda K., Yagita H., Smyth M.J. (2002). Induction of tumor-specific T cell memory by NK cell-mediated tumor rejection. Nat. Immunol..

[33] Zhang Q., Bi J., Zheng X., Chen Y., Wang H., Wu W., Wang Z., Wu Q., Peng H., Wei H. (2018). Blockade of the checkpoint receptor TIGIT prevents NK cell exhaustion and elicits potent anti-tumor immunity. Nat. Immunol..

[34] Finisguerra V., Di Conza G., Di Matteo M., Serneels J., Costa S., Thompson A.A.R., Wauters E., Walmsley S., Prenen H., Granot Z. (2015). MET is required for the recruitment of anti-tumoural neutrophils. Nature.

[35] Showalter A., Limaye A., Oyer J.L., Igarashi R., Kittipatarin C., Copik A.J., Khaled A.R. (2017). Cytokines in immunogenic cell death: Applications for cancer immunotherapy. Cytokine.

[36] Böttcher J.P., Bonavita E., Chakravarty P., Blees H., Cabeza-Cabrerizo M., Sammicheli S., Rogers N.C., Sahai E., Zelenay S., Reis e Sousa C. (2018). NK cells stimulate recruitment of cDC1 into the tumor microenvironment promoting cancer immune control. Cell.

[37] Tran Janco J.M., Lamichhane P., Karyampudi L., Knutson K.L. (2015). Tumor-infiltrating dendritic cells in cancer pathogenesis. J. Immunol..

[38] Mildner A., Jung S. (2014). Development and function of dendritic cell subsets. Immunity.

[39] Walzer T., Dalod M., Robbins S.H., Zitvogel L., Vivier E. (2005). Natural-killer cells and dendritic cells: “L’union fait la force”. Blood.

[40] Mantovani A., Sica A. (2010). Macrophages, innate immunity and cancer: Balance, tolerance, and diversity. Curr. Opin. Immunol..

[41] Brown J.M., Recht L., Strober S. (2017). The promise of targeting macrophages in cancer therapy. Clin. Cancer Res..

[42] Quail D.F., Joyce J.A. (2013). Microenvironmental regulation of tumor progression and metastasis. Nat. Med..

[43] Diefenbach A., Jamieson A.M., Liu S.D., Shastri N., Raulet D.H. (2000). Ligands for the murine NKG2D receptor: Expression by tumor cells and activation of NK cells and macrophages. Nat. Immunol..

[44] Miguel R.D.V., Cherpes T.L., Watson L.J., McKenna K.C. (2010). CTL induction of tumoricidal nitric oxide production by intratumoral macrophages is critical for tumor elimination. J. Immunol..

[45] Pantano F., Berti P., Guida F.M., Perrone G., Vincenzi B., Amato M.M.C., Righi D., Dell’Aquila E., Graziano F., Catalano V. (2013). The role of macrophages polarization in predicting prognosis of radically resected gastric cancer patients. J. Cell. Mol. Med..

[46] Mantovani A., Sozzani S., Locati M., Allavena P., Sica A. (2002). Macrophage polarization: Tumor-associated macrophages as a paradigm for polarized M2 mononuclear phagocytes. Trends Immunol..

[47] Fridlender Z.G., Sun J., Kim S., Kapoor V., Cheng G., Ling L., Worthen G.S., Albelda S.M. (2009). Polarization of Tumor-Associated Neutrophil Phenotype by TGF-β: “N1” versus “N2” TAN. Cancer Cell.

[48] Andzinski L., Kasnitz N., Stahnke S., Wu C.F., Gereke M., Von Köckritz-Blickwede M., Schilling B., Brandau S., Weiss S., Jablonska J. (2016). Type I IFNs induce anti-tumor polarization of tumor associated neutrophils in mice and human. Int. J. Cancer.

[49] Bonde A.K., Tischler V., Kumar S., Soltermann A., Schwendener R.A. (2012). Intratumoral macrophages contribute to epithelial-mesenchymal transition in solid tumors. BMC Cancer.

[50] Bao S., Wu Q., McLendon R.E., Hao Y., Shi Q., Hjelmeland A.B., Dewhirst M.W., Bigner D.D., Rich J.N. (2006). Glioma stem cells promote radioresistance by preferential activation of the DNA damage response. Nature.

[51] Blanpain C., Mohrin M., Sotiropoulou P.A., Passegué E. (2011). DNA-damage response in tissue-specific and cancer stem cells. Cell Stem. Cell.

[52] Begicevic R.R., Falasca M. (2017). ABC transporters in cancer stem cells: Beyond chemoresistance. Int. J. Mol. Sci..

[53] El Khoury F., Corcos L., Durand S., Simon B., Le Jossic-Corcos C. (2016). Acquisition of anticancer drug resistance is partially associated with cancer stemness in human colon cancer cells. Int. J. Oncol..

[54] Fletcher J.I., Haber M., Henderson M.J., Norris M.D. (2010). ABC transporters in cancer: More than just drug efflux pumps. Nat. Rev. Cancer.

[55] Di Tomaso T., Mazzoleni S., Wang E., Sovena G., Clavenna D., Franzin A., Mortini P., Ferrone S., Doglioni C., Marincola F.M. (2010). Immunobiological characterization of cancer stem cells isolated from glioblastoma patients. Clin. Cancer Res..

[56] Schatton T., Schütte U., Frank N.Y., Zhan Q., Hoerning A., Robles S.C., Zhou J., Hodi F.S., Spagnoli G.C., Murphy G.F. (2010). Modulation of T-cell activation by malignant melanoma initiating cells. Cancer Res..

[57] Volonté A., Di Tomaso T., Spinelli M., Todaro M., Sanvito F., Albarello L., Bissolati M., Ghirardelli L., Orsenigo E., Ferrone S. (2014). Cancer-initiating cells from colorectal cancer patients escape from T cell–mediated immunosurveillance in vitro through membrane-bound IL-4. J. Immunol..

[58] Grau J.J., Mesía R., De La Iglesia-Vicente M., Williams E.S., Taberna M., Caballero M., Larque A.B., De La Oliva J., Cordón-Cardo C., Domingo-Domenech J. (2016). Enrichment of cells with cancer stem cell-like markers in relapses of chemoresistant patients with locally advanced head and neck squamous cell carcinoma. Oncology.

[59] MacCalli C., Volontè A., Cimminiello C., Parmiani G. (2014). Immunology of cancer stem cells in solid tumours. A review. Eur. J. Cancer.

[60] Maccalli C., Rasul K.I., Elawad M., Ferrone S. (2018). The role of cancer stem cells in the modulation of anti-tumor immune responses. Semin. Cancer Biol..

[61] Morrison B.J., Steel J.C., Morris J.C. (2018). Reduction of MHC-I expression limits T-lymphocyte-mediated killing of Cancer-initiating cells. BMC Cancer.

[62] Abdullah L.N., Chow E.K.-H. (2013). Mechanisms of chemoresistance in cancer stem cells. Clin. Transl. Med..

[63] Ni J., Cozzi P., Hao J., Duan W., Graham P., Kearsley J., Li Y. (2014). Cancer stem cells in prostate cancer chemoresistance. Curr. Cancer Drug Targets.

[64] Qadir A.S., Ceppi P., Brockway S., Law C., Mu L., Khodarev N.N., Kim J., Zhao J.C., Putzbach W., Murmann A.E. (2017). CD95/fas increases stemness in cancer cells by inducing a STAT1-dependent type I interferon response. Cell Rep..

[65] Ceppi P., Hadji A., Kohlhapp F.J., Pattanayak A., Hau A., Liu X., Liu H., Murmann A.E., Peter M.E. (2014). CD95 and CD95L promote and protect cancer stem cells. Nat. Commun..

[66] Shang B., Liu Y., Jiang S.J., Liu Y. (2015). Prognostic value of tumor-infiltrating FoxP3+ regulatory T cells in cancers: A systematic review and meta-analysis. Sci. Rep..

[67] Wang B., Wang Q., Wang Z., Jiang J., Yu S.C., Ping Y.F., Yang J., Xu S.L., Ye X.Z., Xu C. (2014). Metastatic consequences of immune escape from NK cell cytotoxicity by human breast cancer stem cells. Cancer Res..

[68] Drukker M., Katz G., Urbach A., Schuldiner M., Markel G., Itskovitz-Eldor J., Reubinoff B., Mandelboim O., Benvenisty N. (2002). Characterization of the expression of MHC proteins in human embryonic stem cells. Proc. Natl. Acad. Sci. USA.

[69] Reim F., Dombrowski Y., Ritter C., Buttmann M., Häusler S., Ossadnik M., Krockenberger M., Beier D., Beier C.P., Dietl J. (2009). Immunoselection of breast and ovarian cancer cells with trastuzumab and natural killer cells: Selective escape of CD44high/CD24 low/HER2low breast cancer stem cells. Cancer Res..

[70] Liu L., Zhang L., Yang L., Li H., Li R., Yu J., Yang L., Wei F., Yan C., Sun Q. (2017). Anti-CD47 antibody as a targeted therapeutic agent for human lung cancer and cancer stem cells. Front. Immunol..

[71] Cioffi M., Trabulo S., Hidalgo M., Costello E., Greenhalf W., Erkan M., Kleeff J., Sainz B., Heeschen C. (2015). Inhibition of CD47 Effectively targets pancreatic cancer stem cells via dual mechanisms. Clin. Cancer Res..

[72] Lee T.K.W., Cheung V.C.H., Lu P., Lau E.Y.T., Ma S., Tang K.H., Tong M., Lo J., Ng I.O.L. (2014). Blockade of CD47-mediated cathepsin S/protease-activated receptor 2 signaling provides a therapeutic target for hepatocellular carcinoma. Hepatology.

[73] Uger R., Johnson L. (2020). Blockade of the CD47-SIRPα axis: A promising approach for cancer immunotherapy. Expert Opin. Biol. Ther..

[74] Ravindran S., Rasool S., Maccalli C. (2019). The cross talk between cancer stem cells/cancer initiating cells and tumor microenvironment: The missing piece of the puzzle for the efficient targeting of these cells with immunotherapy. Cancer Microenviron..

[75] Inoda S., Hirohashi Y., Torigoe T., Morita R., Takahashi A., Asanuma H., Nakatsugawa M., Nishizawa S., Tamura Y., Tsuruma T. (2011). Cytotoxic T lymphocytes efficiently recognize human colon cancer stem-like cells. Am. J. Pathol..

[76] Chikamatsu K., Takahashi G., Sakakura K., Ferrone S., Masuyama K. (2011). Immunoregulatory properties of CD44+ cancer stem-like cells in squamous cell carcinoma of the head and neck. Head Neck.

[77] Ames E., Canter R.J., Grossenbacher S.K., Mac S., Chen M., Smith R.C., Hagino T., Perez-Cunningham J., Sckisel G.D., Urayama S. (2015). NK cells preferentially target tumor cells with a cancer stem cell phenotype. J. Immunol..

[78] Shlush L.I., Chapal-Ilani N., Adar R., Pery N., Maruvka Y., Spiro A., Shouval R., Rowe J.M., Tzukerman M., Bercovich D. (2012). Cell lineage analysis of acute leukemia relapse uncovers the role of replication-rate heterogeneity and microsatellite instability. Blood.

[79] Vahidian F., Duijf P.H.G., Safarzadeh E., Derakhshani A., Baghbanzadeh A., Baradaran B. (2019). Interactions between cancer stem cells, immune system and some environmental components: Friends or foes?. Immunol. Lett..

[80] De Vlaeminck Y., González-Rascón A., Goyvaerts C., Breckpot K. (2016). Cancer-associated myeloid regulatory cells. Front. Immunol..

[81] Mantovani A., Marchesi F., Malesci A., Laghi L., Allavena P. (2017). Tumour-associated macrophages as treatment targets in oncology. Nat. Rev. Clin. Oncol..

[82] Ruffell B., Chang-Strachan D., Chan V., Rosenbusch A., Ho C.M.T., Pryer N., Daniel D., Hwang E.S., Rugo H.S., Coussens L.M. (2014). Macrophage IL-10 blocks CD8+ T cell-dependent responses to chemotherapy by suppressing IL-12 expression in intratumoral dendritic cells. Cancer Cell.

[83] Tauriello D.V.F., Palomo-Ponce S., Stork D., Berenguer-Llergo A., Badia-Ramentol J., Iglesias M., Sevillano M., Ibiza S., Cañellas A., Hernando-Momblona X. (2018). TGFβ drives immune evasion in genetically reconstituted colon cancer metastasis. Nature.

[84] Gabrilovich D.I., Ostrand-Rosenberg S., Bronte V. (2012). Coordinated regulation of myeloid cells by tumours. Nat. Rev. Immunol..

[85] Sica A., Guarneri V., Gennari A. (2019). Myelopoiesis, metabolism and therapy: A crucial crossroads in cancer progression. Cell Stress.

[86] Strauss L., Sangaletti S., Consonni F.M., Szebeni G., Morlacchi S., Totaro M.G., Porta C., Anselmo A., Tartari S., Doni A. (2015). RORC1 regulates tumor-promoting “emergency” granulo-monocytopoiesis. Cancer Cell.

[87] Medina E., Hartl D. (2018). Myeloid-derived suppressor cells in infection: A general overview. J. Innate Immun..

[88] Boettcher S., Manz M.G. (2017). Regulation of inflammation- and infection-driven hematopoiesis. Trends Immunol..

[89] Ueha S., Shand F.H.W., Matsushima K. (2011). Myeloid cell population dynamics in healthy and tumor-bearing mice. Int. Immunopharmacol..

[90] Velten L., Haas S.F., Raffel S., Blaszkiewicz S., Islam S., Hennig B.P., Hirche C., Lutz C., Buss E.C., Nowak D. (2017). Human haematopoietic stem cell lineage commitment is a continuous process. Nat. Cell Biol..

[91] Sica A., Bronte V. (2007). Altered macrophage differentiation and immune dysfunction in tumor development. J. Clin. Invest..

[92] Lau E.Y.T., Ho N.P.Y., Lee T.K.W. (2017). Cancer stem cells and their microenvironment: Biology and therapeutic implications. Stem. Cells Int..

[93] Inamoto S., Itatani Y., Yamamoto T., Minamiguchi S., Hirai H., Iwamoto M., Hasegawa S., Taketo M.M., Sakai Y., Kawada K. (2016). Loss of SMAD4 promotes colorectal cancer progression by accumulation of myeloid-derived suppressor cells through the CCL15-CCR1 chemokine axis. Clin. Cancer Res..

[94] Panni R.Z., Sanford D.E., Belt B.A., Mitchem J.B., Worley L.A., Goetz B.D., Mukherjee P., Wang-Gillam A., Link D.C., Denardo D.G. (2014). Tumor-induced STAT3 activation in monocytic myeloid-derived suppressor cells enhances stemness and mesenchymal properties in human pancreatic cancer. Cancer Immunol. Immunother..

[95] Wang Y., Yin K., Tian J., Xia X., Ma J., Tang X., Xu H., Wang S. (2019). Granulocytic myeloid-derived suppressor cells promote the stemness of colorectal cancer cells through exosomal S100A9. Adv. Sci..

[96] Cui T.X., Kryczek I., Zhao L., Zhao E., Kuick R., Roh M.H., Vatan L., Szeliga W., Mao Y., Thomas D.G. (2013). Myeloid-derived suppressor cells enhance stemness of cancer cells by inducing microRNA101 and suppressing the corepressor CTBP2. Immunity.

[97] Kuroda H., Mabuchi S., Yokoi E., Komura N., Kozasa K., Matsumoto Y., Kawano M., Takahashi R., Sasano T., Shimura K. (2018). Prostaglandin E2 produced by myeloid-derived suppressive cells induces cancer stem cells in uterine cervical cancer. Oncotarget.

[98] Peng D., Tanikawa T., Li W., Zhao L., Vatan L., Szeliga W., Wan S., Wei S., Wang Y., Liu Y. (2016). Myeloid-derived suppressor cells endow stem-like qualities to breast cancer cells through IL6/STAT3 and NO/NOTCH cross-talk signaling. Cancer Res..

[99] Wan S., Zhao E., Kryczek I., Vatan L., Sadovskaya A., Ludema G., Simeone D.M., Zou W., Welling T.H. (2014). Tumor-associated macrophages produce interleukin 6 and signal via STAT3 to promote expansion of human hepatocellular carcinoma stem cells. Gastroenterology.

[100] Jinushi M., Chiba S., Yoshiyama H., Masutomi K., Kinoshita I., Dosaka-Akita H., Yagita H., Takaoka A., Tahara H. (2011). Tumor-associated macrophages regulate tumorigenicity and anticancer drug responses of cancer stem/initiating cells. Proc. Natl. Acad. Sci. USA.

[101] Mitchem J.B., Brennan D.J., Knolhoff B.L., Belt B.A., Zhu Y., Sanford D.E., Belaygorod L., Carpenter D., Collins L., Piwnica-Worms D. (2013). Targeting tumor-infiltrating macrophages decreases tumor-initiating cells, relieves immunosuppression, and improves chemotherapeutic responses. Cancer Res..

[102] Zhou W., Ke S.Q., Huang Z., Flavahan W., Fang X., Paul J., Wu L., Sloan A.E., McLendon R.E., Li X. (2015). Periostin secreted by glioblastoma stem cells recruits M2 tumour-associated macrophages and promotes malignant growth. Nat. Cell Biol..

[103] Wu A., Wei J., Kong L.Y., Wang Y., Priebe W., Qiao W., Sawaya R., Heimberger A.B. (2010). Glioma cancer stem cells induce immunosuppressive macrophages/microglia. Neuro. Oncol..

[104] Zhang Q., Cai D.J., Li B. (2015). Ovarian cancer stem-like cells elicit the polarization of M2 macrophages. Mol. Med. Rep..

[105] Nusblat L.M., Carroll M.J., Roth C.M. (2017). Crosstalk between M2 macrophages and glioma stem cells. Cell. Oncol..

[106] Kokubu Y., Tabu K., Muramatsu N., Wang W., Murota Y., Nobuhisa I., Jinushi M., Taga T. (2016). Induction of protumoral CD11chigh macrophages by glioma cancer stem cells through GM-CSF. Genes to Cells.

[107] Sainz B., Martín B., Tatari M., Heeschen C., Guerra S. (2014). ISG15 is a critical microenvironmental factor for pancreatic cancer stem cells. Cancer Res..

[108] Chen R.H., Du Y., Han P., Wang H.B., Liang F.Y., Feng G.K., Zhou A.J., Cai M.Y., Zhong Q., Zeng M.S. (2016). ISG15 predicts poor prognosis and promotes cancer stem cell phenotype in nasopharyngeal carcinoma. Oncotarget.

[109] Jinushi M., Baghdadi M., Chiba S., Yoshiyama H. (2012). Regulation of cancer stem cell activities by tumor-associated macrophages. Am. J. Cancer Res..

[110] Fan Q.M., Jing Y.Y., Yu G.F., Kou X.R., Ye F., Gao L., Li R., Zhao Q.D., Yang Y., Lu Z.H. (2014). Tumor-associated macrophages promote cancer stem cell-like properties via transforming growth factor-beta1-induced epithelial-mesenchymal transition in hepatocellular carcinoma. Cancer Lett..

[111] Plaks V., Kong N., Werb Z. (2015). The cancer stem cell niche: How essential is the niche in regulating stemness of tumor cells?. Cell Stem Cell.

[112] Elaimy A.L., Guru S., Chang C., Ou J., Amante J.J., Zhu L.J., Goel H.L., Mercurio A.M. (2018). VEGF-neuropilin-2 signaling promotes stem-like traits in breast cancer cells by TAZ-mediated repression of the Rac GAP β2-chimaerin. Sci. Signal..

[113] Mercurio A.M. (2019). VEGF/neuropilin signaling in cancer stem cells. Int. J. Mol. Sci..

[114] Raghavan S., Mehta P., Xie Y., Lei Y.L., Mehta G. (2019). Ovarian cancer stem cells and macrophages reciprocally interact through the WNT pathway to promote pro-tumoral and malignant phenotypes in 3D engineered microenvironments. J. Immunother. Cancer.

[115] Lu H., Clauser K.R., Tam W.L., Fröse J., Ye X., Eaton E.N., Reinhardt F., Donnenberg V.S., Bhargava R., Carr S.A. (2014). A breast cancer stem cell niche supported by juxtacrine signalling from monocytes and macrophages. Nat. Cell Biol..

[116] Gabrusiewicz K., Li X., Wei J., Hashimoto Y., Marisetty A.L., Ott M., Wang F., Hawke D., Yu J., Healy L.M. (2018). Glioblastoma stem cell-derived exosomes induce M2 macrophages and PD-L1 expression on human monocytes. Oncoimmunology.

[117] Domenis R., Cesselli D., Toffoletto B., Bourkoula E., Caponnetto F., Manini I., Beltrami A.P., Ius T., Skrap M., Di Loreto C. (2017). Systemic T cells immunosuppression of glioma stem cell-derived exosomes is mediated by monocytic myeloid-derived suppressor cells. PLoS ONE.

[118] Biswas S., Mandal G., Roy Chowdhury S., Purohit S., Payne K.K., Anadon C., Gupta A., Swanson P., Yu X., Conejo-Garcia J.R. (2019). Exosomes produced by mesenchymal stem cells drive differentiation of myeloid cells into immunosuppressive M2-polarized macrophages in breast cancer. J. Immunol..

[119] Grange C., Tapparo M., Tritta S., Deregibus M.C., Battaglia A., Gontero P., Frea B., Camussi G. (2015). Role of HLA-G and extracellular vesicles in renal cancer stem cell-induced inhibition of dendritic cell differentiation. BMC Cancer.

[120] Zhang B., Yin Y., Lai R.C., Tan S.S., Choo A.B.H., Lim S.K. (2014). Mesenchymal stem cells secrete immunologically active exosomes. Stem. Cells Dev..

[121] Hwang W.L., Lan H.Y., Cheng W.C., Huang S.C., Yang M.H. (2019). Tumor stem-like cell-derived exosomal RNAs prime neutrophils for facilitating tumorigenesis of colon cancer. J. Hematol. Oncol..

[122] Alzahrani F.A., El-Magd M.A., Abdelfattah-Hassan A., Saleh A.A., Saadeldin I.M., El-Shetry E.S., Badawy A.A., Alkarim S. (2018). Potential effect of exosomes derived from cancer stem cells and MSCs on progression of DEN-induced HCC in rats. Stem Cells Int..

[123] Sun Z.P., Li A.Q., Jia W.H., Ye S., Van Eps G., Yu J.M., Yang W.J. (2017). MicroRNA expression profiling in exosomes derived from gastric cancer stem-like cells. Oncotarget.

[124] Grange C., Tapparo M., Collino F., Vitillo L., Damasco C., Deregibus M.C., Tetta C., Bussolati B., Camussi G. (2011). Microvesicles released from human renal cancer stem cells stimulate angiogenesis and formation of lung premetastatic niche. Cancer Res..

[125] Wang L., Yang G., Zhao D., Wang J., Bai Y., Peng Q., Wang H., Fang R., Chen G., Wang Z. (2019). CD103-positive CSC exosome promotes EMT of clear cell renal cell carcinoma: Role of remote MiR-19b-3p. Mol. Cancer.

[126] Hardin H., Helein H., Meyer K., Robertson S., Zhang R., Zhong W., Lloyd R.V. (2018). Thyroid cancer stem-like cell exosomes: Regulation of EMT via transfer of lncRNAs. Lab. Investig..

[127] McAllister S.S., Weinberg R.A. (2014). The tumour-induced systemic environment as a critical regulator of cancer progression and metastasis. Nat. Cell Biol..

[128] Peinado H., Lavotshkin S., Lyden D. (2011). The secreted factors responsible for pre-metastatic niche formation: Old sayings and new thoughts. Semin. Cancer Biol..

[129] Fidler I.J. (2003). The pathogenesis of cancer metastasis: The “seed and soil” hypothesis revisited. Nat. Rev. Cancer.

[130] Kalluri R., Weinberg R.A. (2009). The basics of epithelial-mesenchymal transition. J. Clin. Invest..

[131] Dang H., Ding W., Emerson D., Rountree C.B. (2011). Snail1 induces epithelial-to-mesenchymal transition and tumor initiating stem cell characteristics. BMC Cancer.

[132] Scheel C., Weinberg R.A. (2012). Cancer stem cells and epithelial-mesenchymal transition: Concepts and molecular links. Semin. Cancer Biol..

[133] Peinado H., Quintanilla M., Cano A. (2003). Transforming growth factor β-1 induces Snail transcription factor in epithelial cell lines. Mechanisms for epithelial mesenchymal transitions. J. Biol. Chem..

[134] Feng X.-H., Derynck R. (2005). Specificity and versatility in Tgf-Β signaling through smads. Annu. Rev. Cell Dev. Biol..

[135] Kasahara Y., Shirota H., Umegaki S., Ishioka C. Contribution of Fcγ receptor IIB to creating a suppressive tumor microenvironment in a mouse model. Proceedings of the Abstracts of the 77th Annual Meeting of the Japanese Cancer Association.

[136] Fazilaty H., Gardaneh M., Bahrami T., Salmaninejad A., Behnam B. (2013). Crosstalk between breast cancer stem cells and metastatic niche: Emerging molecular metastasis pathway?. Tumor Biol..

[137] Hiratsuka S., Watanabe A., Aburatani H., Maru Y. (2006). Tumour-mediated upregulation of chemoattractants and recruitment of myeloid cells predetermines lung metastasis. Nat. Cell Biol..

[138] Sceneay J., Chow M.T., Chen A., Halse H.M., Wong C.S.F., Andrews D.M., Sloan E.K., Parker B.S., Bowtell D.D., Smyth M.J. (2012). Primary tumor hypoxia recruits CD11b+/Ly6Cmed/ Ly6G+ immune suppressor cells and compromises NK cell cytotoxicity in the premetastatic niche. Cancer Res..

[139] Liu Y., Cao X. (2016). Characteristics and significance of the pre-metastatic niche. Cancer Cell.

[140] Kitamura T., Qian B.Z., Pollard J.W. (2015). Immune cell promotion of metastasis. Nat. Rev. Immunol..

[141] Kaplan R.N., Riba R.D., Zacharoulis S., Bramley A.H., Vincent L., Costa C., MacDonald D.D., Jin D.K., Shido K., Kerns S.A. (2005). VEGFR1-positive haematopoietic bone marrow progenitors initiate the pre-metastatic niche. Nature.

[142] Zhou Z.N., Sharma V.P., Beaty B.T., Roh-Johnson M., Peterson E.A., Van Rooijen N., Kenny P.A., Wiley H.S., Condeelis J.S., Segall J.E. (2014). Autocrine HBEGF expression promotes breast cancer intravasation, metastasis and macrophage-independent invasion in vivo. Oncogene.

[143] Chen J., Yao Y., Gong C., Yu F., Su S., Chen J., Liu B., Deng H., Wang F., Lin L. (2011). CCL18 from tumor-associated macrophages promotes breast cancer metastasis via PITPNM3. Cancer Cell.

[144] Sangaletti S., Di Carlo E., Gariboldi S., Miotti S., Cappetti B., Parenza M., Rumio C., Brekken R.A., Chiodoni C., Colombo M.P. (2008). Macrophage-derived SPARC bridges tumor cell-extracellular matrix interactions toward metastasis. Cancer Res..

[145] Curiel T.J., Coukos G., Zou L., Alvarez X., Cheng P., Mottram P., Evdemon-Hogan M., Conejo-Garcia J.R., Zhang L., Burow M. (2004). Specific recruitment of regulatory T cells in ovarian carcinoma fosters immune privilege and predicts reduced survival. Nat. Med..

[146] Kenkel J.A., Tseng W.W., Davidson M.G., Tolentino L.L., Choi O., Bhattacharya N., Seeley E.S., Winer D.A., Reticker-Flynn N.E., Engleman E.G. (2017). An immunosuppressive dendritic cell subset accumulates at secondary sites and promotes metastasis in pancreatic cancer. Cancer Res..

[147] Coffelt S.B., Kersten K., Doornebal C.W., Weiden J., Vrijland K., Hau C.S., Verstegen N.J.M., Ciampricotti M., Hawinkels L.J.A.C., Jonkers J. (2015). IL-17-producing γδ T cells and neutrophils conspire to promote breast cancer metastasis. Nature.

[148] Wellenstein M.D., Coffelt S.B., Duits D.E.M., van Miltenburg M.H., Slagter M., de Rink I., Henneman L., Kas S.M., Prekovic S., Hau C.S. (2019). Loss of p53 triggers WNT-dependent systemic inflammation to drive breast cancer metastasis. Nature.

[149] Wculek S.K., Malanchi I. (2015). Neutrophils support lung colonization of metastasis-initiating breast cancer cells. Nature.

[150] Nawaz M., Fatima F., Vallabhaneni K.C., Penfornis P., Valadi H., Ekström K., Kholia S., Whitt J.D., Fernandes J.D., Pochampally R. (2016). Extracellular vesicles: Evolving factors in stem cell biology. Stem Cells Int..

[151] Kong J., Tian H., Zhang F., Zhang Z., Li J., Liu X., Li X., Liu J., Li X., Jin D. (2019). Extracellular vesicles of carcinoma-associated fibroblasts creates a pre-metastatic niche in the lung through activating fibroblasts. Mol. Cancer.

[152] Kalluri R., Zeisberg M. (2006). Fibroblasts in cancer. Nat. Rev. Cancer.

[153] Giannoni E., Bianchini F., Masieri L., Serni S., Torre E., Calorini L., Chiarugi P. (2010). Reciprocal activation of prostate cancer cells and cancer-associated fibroblasts stimulates epithelial-mesenchymal transition and cancer stemness. Cancer Res..

[154] Chen W.J., Ho C.C., Chang Y.L., Chen H.Y., Lin C.A., Ling T.Y., Yu S.L., Yuan S.S., Louisa Chen Y.J., Lin C.Y. (2014). Cancer-associated fibroblasts regulate the plasticity of lung cancer stemness via paracrine signalling. Nat. Commun..

[155] Costa A., Kieffer Y., Scholer-Dahirel A., Pelon F., Bourachot B., Cardon M., Sirven P., Magagna I., Fuhrmann L., Bernard C. (2018). Fibroblast heterogeneity and immunosuppressive environment in human breast cancer. Cancer Cell.

[156] Hu Y., Yan C., Mu L., Huang K., Li X., Tao D., Wu Y., Qin J. (2015). Fibroblast-derived exosomes contribute to chemoresistance through priming cancer stem cells in colorectal cancer. PLoS ONE.

[157] Boelens M.C., Wu T.J., Nabet B.Y., Xu B., Qiu Y., Yoon T., Azzam D.J., Twyman-Saint Victor C., Wiemann B.Z., Ishwaran H. (2014). Exosome transfer from stromal to breast cancer cells regulates therapy resistance pathways. Cell.

[158] Su S., Chen J., Yao H., Liu J., Yu S., Lao L., Wang M., Luo M., Xing Y., Chen F. (2018). CD10+GPR77+ cancer-associated fibroblasts promote cancer formation and chemoresistance by sustaining cancer stemness. Cell.

[159] Donnarumma E., Fiore D., Nappa M., Roscigno G., Adamo A., Iaboni M., Russo V., Affinito A., Puoti I., Quintavalle C. (2017). Cancer-associated fibroblasts release exosomal microRNAs that dictate an aggressive phenotype in breast cancer. Oncotarget.

[160] Luga V., Zhang L., Viloria-Petit A.M., Ogunjimi A.A., Inanlou M.R., Chiu E., Buchanan M., Hosein A.N., Basik M., Wrana J.L. (2012). Exosomes mediate stromal mobilization of autocrine Wnt-PCP signaling in breast cancer cell migration. Cell.

[161] Malanchi I., Santamaria-Martínez A., Susanto E., Peng H., Lehr H.A., Delaloye J.F., Huelsken J. (2012). Interactions between cancer stem cells and their niche govern metastatic colonization. Nature.

[162] Fessler E., Dijkgraaf F.E., De Sousa E., Melo F., Medema J.P. (2013). Cancer stem cell dynamics in tumor progression and metastasis: Is the microenvironment to blame?. Cancer Lett..

[163] Oskarsson T., Acharyya S., Zhang X.H.F., Vanharanta S., Tavazoie S.F., Morris P.G., Downey R.J., Manova-Todorova K., Brogi E., Massagué J. (2011). Breast cancer cells produce tenascin C as a metastatic niche component to colonize the lungs. Nat. Med..

[164] Lytle N.K., Barber A.G., Reya T. (2018). Stem cell fate in cancer growth, progression and therapy resistance. Nat. Rev. Cancer.

[165] Jiang W., Chan C.K., Weissman I.L., Kim B.Y.S., Hahn S.M. (2016). Immune priming of the tumor microenvironment by radiation. Trends Cancer.

[166] Frey B., Rückert M., Deloch L., Rühle P.F., Derer A., Fietkau R., Gaipl U.S. (2017). Immunomodulation by ionizing radiation—Impact for design of radio-immunotherapies and for treatment of inflammatory diseases. Immunol. Rev..

[167] Jarosz-Biej M., Smolarczyk R., Cichoń T., Kułach N. (2019). Tumor microenvironment as a “game changer” in cancer radiotherapy. Int. J. Mol. Sci..

[168] Holley A.K., Miao L., St. Clair D.K., St. Clair W.H. (2014). Redox-modulated phenomena and radiation therapy: The central role of superoxide dismutases. Antioxidants Redox Signal..

[169] Kuonen F., Secondini C., Rüegg C. (2012). Molecular pathways: Emerging pathways mediating growth, invasion, and metastasis of tumors progressing in an irradiated microenvironment. Clin. Cancer Res..

[170] Shigdar S., Lin J., Li Y., Yang C.J., Wei M., Zhu Y., Liu H., Duan W. (2012). Cancer stem cell targeting: The next generation of cancer therapy and molecular imaging. Ther. Deliv..

[171] MacDonagh L., Gray S.G., Breen E., Cuffe S., Finn S.P., O’Byrne K.J., Barr M.P. (2018). BBI608 inhibits cancer stemness and reverses cisplatin resistance in NSCLC. Cancer Lett..

[172] Wong A.L., Soo R.A., Tan D.S., Lee S.C., Lim J.S., Marban P.C., Kong L.R., Lee Y.J., Wang L.Z., Thuya W.L. (2015). Phase I and biomarker study of OPB-51602, a novel signal transducer and activator of transcription (STAT) 3 inhibitor, in patients with refractory solid malignancies. Ann. Oncol..

[173] Han D., Yu T., Dong N., Wang B., Sun F., Jiang D. (2019). Napabucasin, a novel STAT3 inhibitor suppresses proliferation, invasion and stemness of glioblastoma cells. J. Exp. Clin. Cancer Res..

[174] Sikic B.I., Lakhani N., Patnaik A., Shah S.A., Chandana S.R., Rasco D., Colevas A.D., O’Rourke T., Narayanan S., Papadopoulos K. (2019). First-in-human, first-in-class phase i trial of the anti-CD47 antibody Hu5F9-G4 in patients with advanced cancers. J. Clin. Oncol..

[175] Zhi Y., Mou Z., Chen J., He Y., Dong H., Fu X., Wu Y. (2015). B7H1 expression and epithelial-to-mesenchymal transition phenotypes on colorectal cancer stem-like cells. PLoS ONE.

[176] Yang Y., Wu K., Zhao E., Li W., Shi L., Xie G., Jiang B., Wang Y., Li R., Zhang P. (2015). B7-H1 enhances proliferation ability of gastric cancer stem-like cells as a receptor. Oncol. Lett..

[177] Gupta H.B., Hurez V., Clark C.A., Sareddy G.R., Vadlamudi R., Li R., Curiel T.J. (2016). Tumor B7-H1 regulates cancer stem cell generation and virulence. J. Immunol..

[178] Chen Y., Di C., Zhang X., Wang J., Wang F., Yan J.F., Xu C., Zhang J., Zhang Q., Li H. (2020). Transforming growth factor β signaling pathway: A promising therapeutic target for cancer. J. Cell. Physiol..

[179] Chen D.S., Hurwitz H. (2018). Combinations of bevacizumab with cancer immunotherapy. Cancer J..

[180] Xu Q., Liu G., Yuan X., Xu M., Wang H., Ji J., Konda B., Black K.L., Yu J.S. (2009). Antigen-specific T-cell response from dendritic cell vaccination using cancer stem-like cell-associated antigens. Stem Cells.

[181] Fesnak A.D., June C.H., Levine B.L. (2016). Engineered T cells: The promise and challenges of cancer immunotherapy. Nat. Rev. Cancer.

[182] Guo Y., Feng K., Wang Y., Han W. (2018). Targeting cancer stem cells by using chimeric antigen receptor-modified T cells: A potential and curable approach for cancer treatment. Protein Cell.

[183] Weiner G.J. (2015). Building better monoclonal antibody-based therapeutics. Nat. Rev. Cancer.

[184] Deng Z., Wu Y., Ma W., Zhang S., Zhang Y.Q. (2015). Adoptive T-cell therapy of prostate cancer targeting the cancer stem cell antigen EpCAM. BMC Immunol..

